# Supplementary material for: Accelerated growth increases the somatic epimutation rate in trees
Source: Nat Commun. 2025 Oct 27;16:9483. doi: 10.1038/s41467-025-65404-9 (PMC12559280; doi:10.1038/s41467-025-65404-9)
Supplement: Supplementary file 1 — Supplementary Information [file 41467_2025_65404_MOESM1_ESM.pdf]

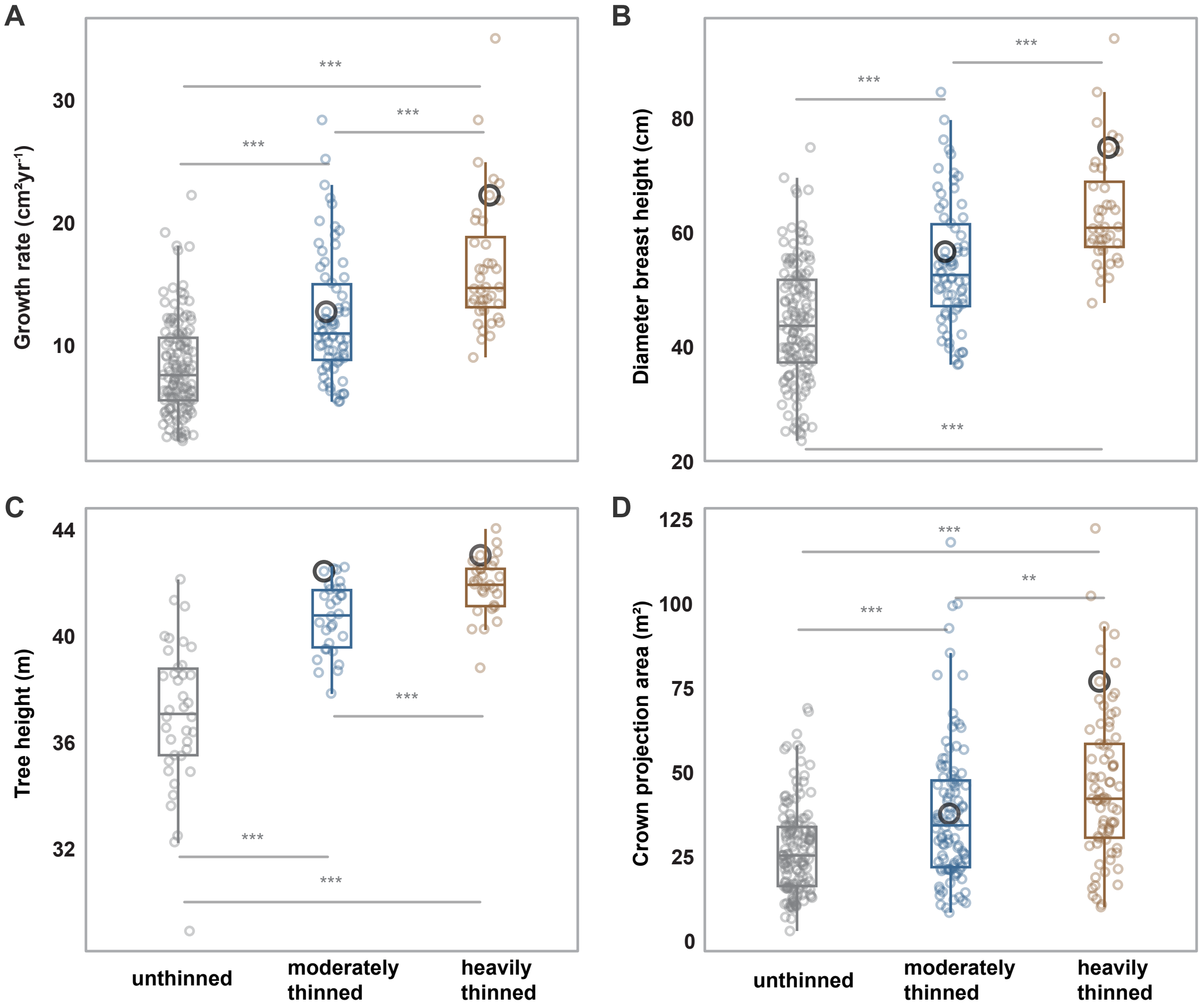

Supplementary Figure 1: Measurement of tree growth parameters. Comparison of growth rate (**A**), diameter at breast height (DBH) (**B**), tree height (**C**), and crown projection area (**D**) under different thinning intensities. All measurements were taken during the 2020 survey, except for crown projection area (**D**), which is based on the 2000 survey—the last occasion when crown projections were recorded for all trees. In 2020, crown projection areas were remeasured for the two felled representative trees, showing increased values: tree 109 (moderately thinned) = 36 m^2; tree 171 (heavily thinned) = 102 m^2. The two felled representative trees are highlighted by dark circles in all subplots. Statistical significance was assessed using a pairwise Wilcoxon rank-sum test with Bonferroni correction for multiple comparisons. Full statistical results are provided in Supplementary Data 5. $^{**}P < 0.01$; $^{***}P < 0.001$. Source data are provided as a Source Data file.

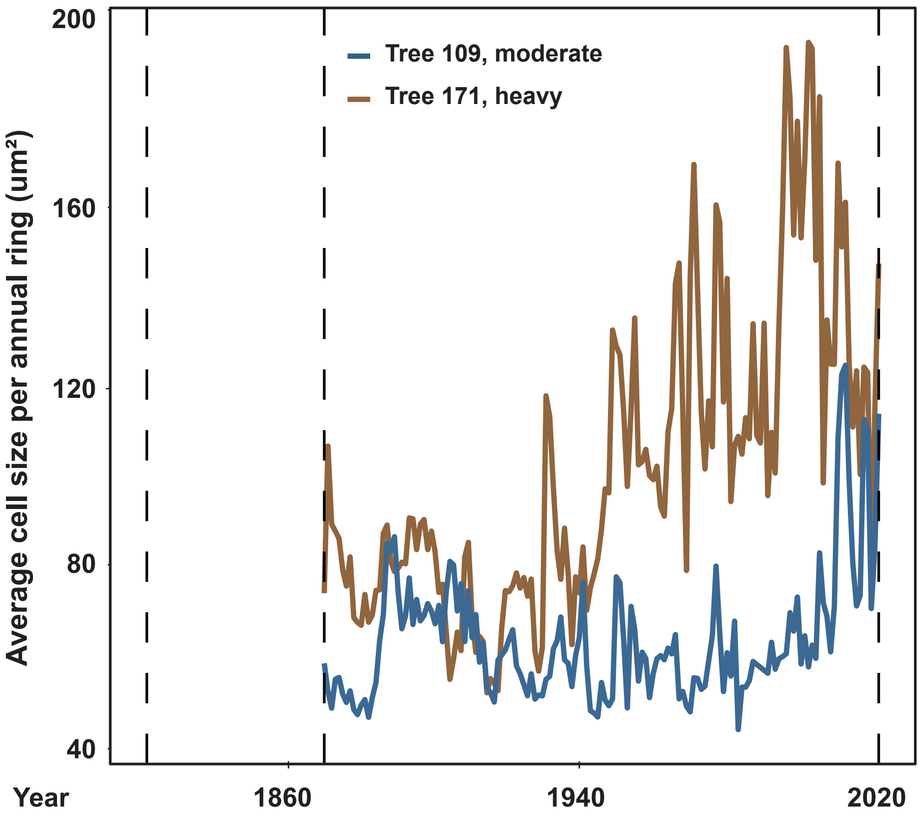

Supplementary Figure 2: Average cell size per annual ring. Tree 171 had significantly larger cells per annual ring than tree 109. Source Data are provided as a Source Data file.

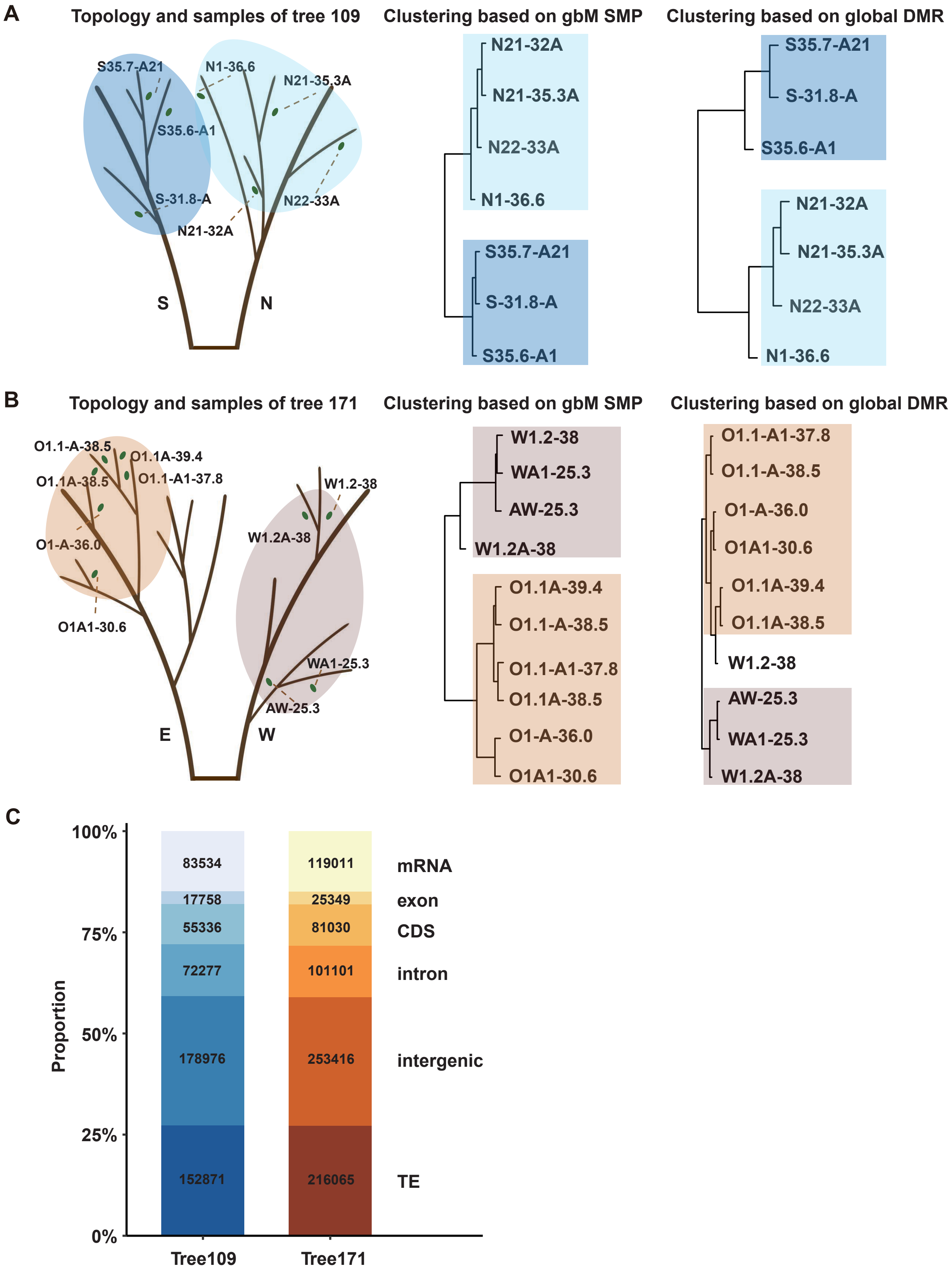

Supplementary Figure 3: DNA methylation-based clustering of leaf samples recapitulates known tree topology. (**A,B**) Sample locations of leaves within the branching topologies of tree 109 and tree 171. Unsupervised sample clustering based on detected SMPs on gbM genes and DMR recapitulates the branching topologies of each of the two trees. Shaded areas indicate the two branches of clustering, enclosing the corresponding samples. (**C**) Distribution of CG-DMRs across different genomic features in tree 109 and tree 171. Overall, Tree 171 exhibits a higher number of CG-context DMRs compared to tree 109, yet both share a similar distribution pattern across genomic regions.

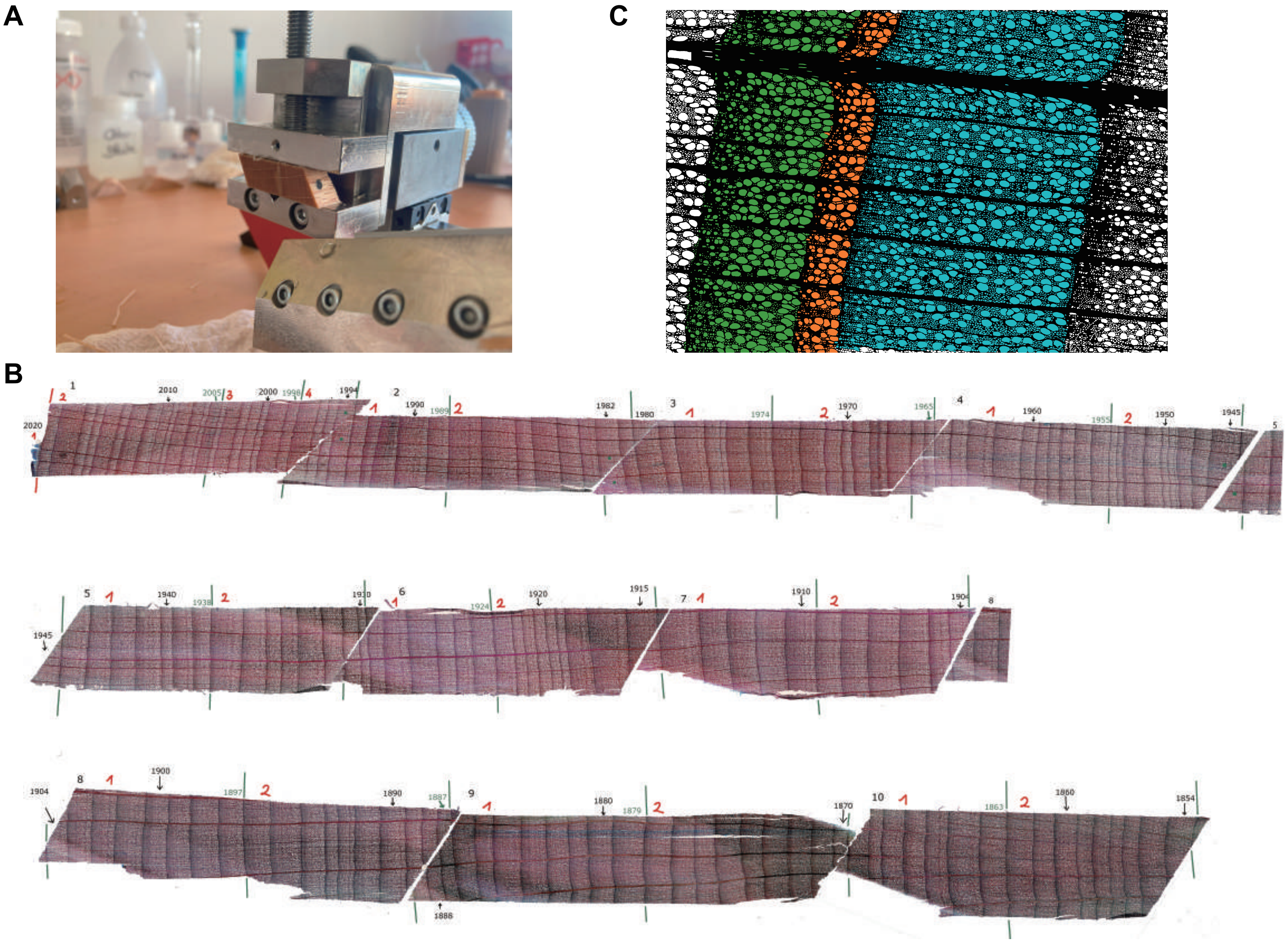

Supplementary Figure 4: Overview of the wood anatomical analysis procedure. First, microslides of small wood segments across the stem, from bark to pith, were taken from both trees (**A**). After preparation of the microslides (staining, embedding, ...), all segments were digitalized with a digital microscope, stitched together, and cross-dated (**B**). Finally, the micrographs were analysed using the DCNN algorithm CARROT to recognise and segment cell types (**C**). The different colours in (**C**) refer to fully identified and separate tree rings. Each highlighted cell is measured and counted.
