# Supplementary material for: Accelerated growth increases the somatic epimutation rate in trees
Source: Nat Commun. 2025 Oct 27;16:9483. doi: 10.1038/s41467-025-65404-9 (PMC12559280; doi:10.1038/s41467-025-65404-9)
Supplement: Supplementary file 2 — Description of Addtional Supplementary Files [file 41467_2025_65404_MOESM2_ESM.pdf]

Description of Additional Supplementary Files

Supplementary Data 1: Sequencing and mapping summary.

Supplementary Data 2: The DMR matrix.

Supplementary Data 3: Epimutation rates by trees and genomic features.

Supplementary Data 4: Input files used for AlphaBeta analysis to estimate epimutation rate.

Supplementary Data 5: Complete results of all statistical tests.
