# Supplementary material for: Accelerated growth increases the somatic epimutation rate in trees
Source: Nat Commun. 2025 Oct 27;16:9483. doi: 10.1038/s41467-025-65404-9 (PMC12559280; doi:10.1038/s41467-025-65404-9)
Supplement: Supplementary file 8 — Reporting Summary [file 41467_2025_65404_MOESM8_ESM.pdf]

nature portfolio

Corresponding author(s): Prof. Dr. Hans Pretzsch
Prof. Dr. Frank Johannes

Last updated by author(s): Feb 16, 2025

Reporting Summary

Nature Portfolio wishes to improve the reproducibility of the work that we publish. This form provides structure for consistency and transparency in reporting. For further information on Nature Portfolio policies, see our Editorial Policies and the Editorial Policy Checklist.

Statistics

For all statistical analyses, confirm that the following items are present in the figure legend, table legend, main text, or Methods section.

n/a	Confirmed	
☐	☒	The exact sample size (*n*) for each experimental group/condition, given as a discrete number and unit of measurement
☐	☒	A statement on whether measurements were taken from distinct samples or whether the same sample was measured repeatedly
☐	☒	The statistical test(s) used AND whether they are one- or two-sided<br>*Only common tests should be described solely by name; describe more complex techniques in the Methods section.*
☒	☐	A description of all covariates tested
☐	☒	A description of any assumptions or corrections, such as tests of normality and adjustment for multiple comparisons
☐	☒	A full description of the statistical parameters including central tendency (e.g. means) or other basic estimates (e.g. regression coefficient) AND variation (e.g. standard deviation) or associated estimates of uncertainty (e.g. confidence intervals)
☐	☒	For null hypothesis testing, the test statistic (e.g. *F*, *t*, *r*) with confidence intervals, effect sizes, degrees of freedom and *P* value noted<br>*Give P values as exact values whenever suitable.*
☒	☐	For Bayesian analysis, information on the choice of priors and Markov chain Monte Carlo settings
☒	☐	For hierarchical and complex designs, identification of the appropriate level for tests and full reporting of outcomes
☐	☒	Estimates of effect sizes (e.g. Cohen's *d*, Pearson's *r*), indicating how they were calculated

Our web collection on statistics for biologists contains articles on many of the points above.

Software and code

Policy information about availability of computer code

Data collection	No custom software was used.
Data analysis	CARROT was employed to recognize and segment all types of cells for each ring from the obtained micrographs: https://github.com/alexander-g/CARROT<br>MethylStar pipeline was employed to process WGBS data: https://github.com/jlab-code/MethylStar<br>jDMRgrid package was used for DMR/SMP calling: https://github.com/jlab-code/jDMRgrid<br>AlphaBeta package was used for epimutation rate estimation: https://github.com/jlab-code/AlphaBeta<br>gbMine was used for identifying gene body methylated genes: https://github.com/jlab-code/gbMine

For manuscripts utilizing custom algorithms or software that are central to the research but not yet described in published literature, software must be made available to editors and reviewers. We strongly encourage code deposition in a community repository (e.g. GitHub). See the Nature Portfolio guidelines for submitting code & software for further information.

Data

Policy information about availability of data

All manuscripts must include a data availability statement. This statement should provide the following information, where applicable:

- Accession codes, unique identifiers, or web links for publicly available datasets
- A description of any restrictions on data availability
- For clinical datasets or third party data, please ensure that the statement adheres to our policy

All WGBS raw data used for this article have been deposited in the NCBI SRA under accession PRJNA1215776, https://www.ncbi.nlm.nih.gov/sra/?term=PRJNA1215776. The source data underlying Fig. 1-4 and Supplementary Fig. 1 are provided as a Source Data file. Source data are provided with this paper.

Research involving human participants, their data, or biological material

Policy information about studies with human participants or human data. See also policy information about sex, gender (identity/presentation), and sexual orientation and race, ethnicity and racism.

Reporting on sex and gender	This study focuses on plant samples and does not involve human participants or human data.
Reporting on race, ethnicity, or other socially relevant groupings	This study focuses on plant samples and does not involve human participants or human data.
Population characteristics	This study focuses on plant samples and does not involve human participants or human data.
Recruitment	This study focuses on plant samples and does not involve human participants or human data.
Ethics oversight	This study focuses on plant samples and does not involve human participants or human data.

Note that full information on the approval of the study protocol must also be provided in the manuscript.

Field-specific reporting

Please select the one below that is the best fit for your research. If you are not sure, read the appropriate sections before making your selection.

☐ Life sciences ☐ Behavioural & social sciences ☒ Ecological, evolutionary & environmental sciences

For a reference copy of the document with all sections, see nature.com/documents/nr-reporting-summary-flat.pdf

Ecological, evolutionary & environmental sciences study design

All studies must disclose on these points even when the disclosure is negative.

Study description	We relied on the long-term thinning trial Fabrikschleichach 15 (FAB 15) in a European beech (Fagus sylvatica L.) forest in Central Europe to obtain trees with exactly the same age but with strongly divergent growth rates. Two representative trees from moderately thinned and heavily thinned plots were selected to estimate epimutation rate. In addition, we randomly selected four trees from moderately thinned plot and three trees from heavily thinned plot as replicates to validate our finding.
Research sample	The beech forest (Fagus sylvatica L.), now over 200 years old, originated from a shelterwood cutting in 1822, and the various treatments and continuous measurements began in 1870/1871. The two selected trees, Tree 109 and Tree 171, are representative individuals of their respective treatments. While the exact genotypes of Tree 109 and Tree 171 have not yet been determined, there is no indication of artificial selection or planting, and all trees in the plots are presumed to stem from the same local gene pool. Therefore, although we cannot confirm a close genetic relationship between the two selected trees, it is reasonable to assume they are representative members of a naturally regenerated, locally adapted beech population subjected to the respective long-term thinning treatments.
Sampling strategy	We selected a representative, average sample tree from each thinned plot for further analyses, which was felled and precisely measured. Both selected trees had a particularly low branching point to allow for distinct differences in branch divergence time within the same tree. After felling, branches along the crown periphery were selected (distributed across lower, middle, and upper crown) and leaves were sampled from the ends of these branches. We followed the sampling methods described in several published studies to collect cambium samples at 1.3 m height from moderately thinned and heavily thinned plots. All samples were immediately stored at -80 °C for DNA extraction.
Data collection	Trees were felled for detailed analyses. After felling, branches along the crown periphery were selected (distributed across lower, middle, and upper crown) and leaves were sampled from the ends of these branches. All leaf samples were kept frozen at -80 °C for subsequent DNA methylation analysis. Additionally, cambium samples were obtained from additional trees (four trees from the moderately thinned and three from the heavily thinned plot). Stem and branch disks were obtained from different positions of the felled trees along their stem axis and crown topology to allow for age, stem growth rate, and cell production estimations.
Timing and spatial scale	All samples were collected in summer 2020, immediately after tree felling. The resulting dendrochronological data span 200 years, covering the onset of thinning treatments in 1870/1871. The site is a representative example of European beech forest for the entire

	region.
Data exclusions	We were supposed to use 21 WGBS data from the cambium samples, but 3 WGBS data were removed from the analysis due to library preparation failure.
Reproducibility	Confirmed. We estimated epimutation rates for two trees from two plots, and then further validated our results with replicated trees.
Randomization	The leaves were randomly selected to provide a broad representation of the three-dimensional branching architecture of each tree for epimutation rate estimation. The cambiums were selected randomly at diameter breast height.
Blinding	Our samples were collected from a long-term field experiment, where we knew that the samples came from three different treatment groups at the time of collection. However, in theory, the study was designed to be blinded. The trees from which the samples were taken were randomly selected from a large population of trees, and the allocation to the groups was not determined during the sample collection process.

Did the study involve field work? ☒ Yes ☐ No

Field work, collection and transport

Field conditions	The natural vegetation in this region are submontane European beech-sessile oak forests. Soils are mainly Loamic Luvisols with sandstone as basic material. The region is characterized by a mild climate with an average annual temperature of 7.5 °C and an annual precipitation of 820 mm.
Location	The forest site is located in the Steigerwald forest. A beech forest in northern Bavaria, Germany (10.62°E longitude and 49.95°N latitude).
Access & import/export	The experimental site Fabrikschleichach 15 (FAB15) is one of the oldest forest research sites in the world and forms part of a network of long-term experimental forests. It belongs to the Bavarian State Forests (BaySF), specifically the Forest Enterprise Ebrach, which granted access for tree felling and subsequent analyses.
Disturbance	Trees were felled within the forest for the purpose of analysis. The felling formed part of the site's regular management and was authorized by the responsible Forest Enterprise of Ebrach. Apart from the tree removal, the forest remained undisturbed.

Reporting for specific materials, systems and methods

We require information from authors about some types of materials, experimental systems and methods used in many studies. Here, indicate whether each material, system or method listed is relevant to your study. If you are not sure if a list item applies to your research, read the appropriate section before selecting a response.

Materials & experimental systems

n/a	Involved in the study
☒	☐ Antibodies
☒	☐ Eukaryotic cell lines
☒	☐ Palaeontology and archaeology
☒	☐ Animals and other organisms
☒	☐ Clinical data
☒	☐ Dual use research of concern
☐	☒ Plants

Methods

n/a	Involved in the study
☒	☐ ChIP-seq
☒	☐ Flow cytometry
☒	☐ MRI-based neuroimaging

Plants

Seed stocks	This study relied on the long-term thinning trial Fabrikschleichach 15 (FAB 15) in a European beech (Fagus sylvatica L.) forest in Central (Germany: 49.926085,10.571165) to obtain trees. Trees were felled in June 2020 and samples were taken.
Novel plant genotypes	The tree is European beech (Fagus sylvatica L.), and no specific genotypes.
Authentication	This study focuses on mature plant tissues collected from a long-term field experiment; no novel seed stock or genotype authentication was required.
